# Future Directions in the Diagnosis and Treatment of APDS and IEI: a Survey of German IEI Centers

**DOI:** 10.3389/fimmu.2023.1279652

**Published:** 2023-10-05

**Authors:** Sven Vanselow, Leif Hanitsch, Fabian Hauck, Julia Körholz, Maria-Elena Maccari, Andrea Meinhardt, Georgios Sogkas, Catharina Schuetz, Bodo Grimbacher

**Affiliations:** ^1^ Infill Healthcare Communication, Königswinter, Germany; ^2^ Institute of Medical Immunology, Institute of Occupational Medicine, Charité – University Medicine Berlin, corporate member of Freie University, Berlin and Humboldt-University of Berlin, Berlin, Germany; ^3^ Department of Pediatric Immunology and Rheumatology, Dr. Von Hauner Children’s Hospital, Ludwig-Maximilians-Universität (LMU) Munich University Hospital, Munich, Germany; ^4^ Department of Pediatrics, University Hospital Carl Gustav Carus, Dresden, Germany; ^5^ University Center for Rare Diseases, University Hospital Carl Gustav Carus, Dresden, Germany; ^6^ Center for Chronic Immunodeficiency, University of Freiburg Medical Center, Freiburg, Germany; ^7^ Division of Pediatric Hematology and Oncology, Department of Pediatrics and Adolescent Medicine, University Medical Center Freiburg, Freiburg, Germany; ^8^ Center for Pediatrics and Adolescent Medicine, Department of Pediatric Oncology, Hematology and Immunodeficiencies, University Hospital Giessen, Giessen, Germany; ^9^ Clinic for Rheumatology and Immunology, Center for Internal Medicine, Hannover Medical School, Hannover, Germany; ^10^ Hannover Medical School, Cluster of Excellence RESIST (EXC 2155), Hannover, Germany

**Keywords:** PID, IEI, APDS, immunodeficiency, stem cell transplantation, HSCT, survey, targeted therapy

## Abstract

**Introduction:**

The diagnosis and treatment of inborn errors of immunity (IEI) is a major challenge as the individual conditions are rare and often characterized by a variety of symptoms, which are often non disease-specific. Ideally, patients are treated in dedicated centers by physicians who specialize in the management of primary immune disorders. In this study, we used the example of Activated PI3Kδ syndrome (APDS), a rare IEI with an estimated prevalence of 1:1,000,000. We conducted surveys by questionnaire and interviewed physicians at different IEI centers in Germany.

**Methods:**

We queried structural aspects of IEI care in Germany, diagnostic procedures in IEI care (including molecular diagnostics), distribution of APDS patients, APDS symptoms and severity, treatment algorithms in APDS, the role of stem cell transplantation and targeted therapies in IEI with focus on APDS. We were especially interested in how genetic diagnostics may influence treatment decisions, e.g. with regard to targeted therapies.

**Results/discussion:**

Most centers care for both pediatric and adult patients. A total of 28 APDS patients are currently being treated at the centers we surveyed. Patient journeys vary considerably, as does severity of disease. Genetic diagnosis continues to gain importance - whole genome sequencing is likely to become routine in IEI in the next few years. According to the experts interviewed, stem cell transplantation and - with new molecules being approved - targeted therapies, will gain in importance for the treatment of APDS and IEI in general.

## Introduction

### IEI and rare diseases

Inborn errors of immunity (IEI) are a category of rare diseases, but more common in the population than often thought, and their prevalence is rising (1, 2). This can be mostly attributed to advancements in genetic testing, which has led to the identification of more immunologic illnesses as distinct disease entities. The latest report of the International Union of Immunological Societies (IUIS) lists about 500 individual IEIs (3). Taken together, the proportion of people affected could exceed 1 in 10.000 in the EU (4). Individual monogenetic defects leading to IEI such as APDS are extremely rare. Activated PI3Kδ syndrome (APDS) is a monogenic IEI caused by dominant germline mutations in the PI3Kδ subunit coding genes *PIK3CD* or *PIK3R1*, and occurs at a rate of around one in a million in the general population. In this study we focused on APDS as an example of the difficulties that occur in the diagnosis and treatment of IEI.

### Diagnostic procedures in IEI and APDS

The diagnosis of rare diseases poses several challenges for physicians. First, the symptoms must be classified correctly. The clinical picture of IEI patients is very heterogeneous, as lymphoproliferation, autoimmune diseases, inflammatory changes, and nonspecific complaints (e.g. of the digestive tract) may be the leading symptoms. Infections pathognomonic for known IEI, severe or prolonged infections may or may not be present. Therefore, to assist general practitioners and pediatricians in early diagnosis, warning signs of IEI have been compiled (5). If IEI is suspected, patients should be referred to a specialized center where immunological diagnostics and genetic testing can be performed according to consented guidelines (6–8). As for individuals with suspected APDS, a number of functionally proven gene variants have been compiled from patient data, however two hotspot mutations are reported by all authors: c.3061 G>A (p. E1021K) for APDS1 and c.1425 + 1 G> (A, C, T) (p.434-475del) for APDS2 (9–12). APDS is caused by gain of function (GOF) variants in the *PIK3CD* gene/p110δ protein (APDS1) or loss of function (LOF) mutations in the *PIK3R1* gene/p85α protein (APDS2), which constitute the subunits of the PI3Kδ kinase, a protein complex that is essential for the maturation of T and B lymphocytes and the regulation of their activity (9, 13, 14). Furthermore, LOF variants of the enzyme PTEN, which is an antagonist of PI3Kδ, have also been published to lead to an APDS-like clinical picture (15). In addition to confirmed pathogenic mutations and many non-pathogenic variants, however, there are other variants whose significance to the development of APDS is unknown (variants of unknown significance = VUS). Moreover, not all causative variants may yet have been identified and entered into one of the databases such as OMIM (www.omim.org) or OrphaNET (www.orpha.net). Hence, further research into the link between PI3Kδ pathway dysregulation and genetics must be conducted. The concept of a “genetics first” approach, in which diagnosis via genetic testing is given priority over the clinical and even laboratory phenotype, has in some settings been used quite successfully in the diagnosis of rare diseases (16). However, the same genetic mutation may result in vastly different phenotypes and degrees of severity, hence the need for a unifying and consented diagnostic approach. In the wake of a new era of targeted therapies, treating the underlying pathomechanism rather than addressing symptoms, has become of relevance to the clinician (17, 18). Before considering targeted therapies, however, functional validation of VUS is essential to address limitations of the “genotype first” approach. In the context of APDS and other autosomal dominant IEI, there is currently no way of predicting disease penetrance, expressivity, and severity with genetics alone.

### Disease mechanisms and clinical picture of APDS

GOF mutations of p110δ, the catalytic subunit, and LOF mutations of p85α, the regulatory subunit of PI3Kδ, lead to chronic hyperactivation of PI3Kδ and hence the PI3K/Akt pathway (9, 13, 14). As a result, the maturation of T and B lymphocytes is affected, leading to an abundance of transitional B cells and plasmablasts while the reservoirs of naïve B cells and both naïve CD4+ and CD8+ T cells are depleted (19, 20). Moreover, there is a lack of immunoglobulin class-switching in B cells, reflected in elevated IgM and low IgG levels. With both the humoral response and T-cell response being affected in most patients, APDS is a combined immunodeficiency (CID). As a consequence, patients experience frequent respiratory tract infections (RTI) and chronic viral infections. The RTIs are thought to be the main contributor to progressive lung damage with bronchiectases, which are frequently seen in APDS patients (21). In addition to their immunodeficiency, patients are affected by lymphoproliferative and autoinflammatory disease, including autoimmune cytopenias, arthritis and gastrointestinal (GI) inflammation. Lymphoproliferation often leads to massively enlarged lymph nodes (lymphadenopathy) and organomegaly. APDS significantly increases the risk of malignant lymphoma, which contributes decisively to APDS patients’ lowered life expectancy (11, 12). Many patients also struggle with failure to thrive and short stature. In addition, some patients, especially with APDS2, show syndromic features unrelated to immunodeficiency, similar to SHORT syndrome (short stature, hyperextensibility of joints, and/or inguinal hernia, ocular depression, Rieger anomaly, and teething delay), usually associated with other pathogenic *PIK3R1* variants (22). APDS2 patients may also show problems with neurocognitive development (11, 12).

### Management of APDS patients

Most APDS patients receive symptom-oriented treatments, including immunoglobulin replacement therapy (IRT), prophylactic antibiotics, and immunosuppressants such as rituximab or rapamycin to dampen lymphoproliferation and autoinflammation. However, these options are often insufficient in controlling progressive lung damage and chronic viral infections, or are associated with long-term side effects (11, 12, 23). In contrast, allogeneic hematopoietic stem cell transplantation (HSCT) offers a curative option for all symptoms related to the hematopoietic system (24). The understanding of the molecular mechanism underlying APDS has also fueled the development of leniolisib, a PI3Kδ-specific inhibitor for targeted treatment which has shown long-term efficacy up to 5 years follow-up. Patients on leniolisib have a great benefit with less infection susceptibility and lymphoproliferative disease with excellent tolerance in clinical studies (25, 26). PI3Kδ inhibition offers the chance of addressing consequences of PI3K/Akt pathway hyperactivity in all cells, but the small molecule must be given lifelong or as a bridging therapy to HSCT. As of early 2023, leniolisib has been approved by the FDA and is in the process of market authorization by the European Medicines Agency (EMA) (27).

### Purpose of this study

Due to the rarity and heterogeneity of IEI, diagnosis and treatment of patients requires a high degree of specialization. Accordingly, patient care is more centralized compared to common conditions. In Germany, the Working Group for Pediatric Immunology (API) lists 14 dedicated IEI centers and several clinics with expertise in IEI patient care. We conducted a survey with 15 physicians from these centers and clinics, using a survey with questions relating to the diagnostic and treatment landscape for IEI in general, and specifically APDS. Our aim was to record typical diagnostic algorithms in IEI (including approach to genetic diagnosis and the genotype-first approach), the most frequent symptoms leading to suspicion of APDS, the distribution of APDS patients in Germany, as well as treatment decisions and their rationale.

## Results and discussion

### Participants and awareness for APDS

**Figure 1:**
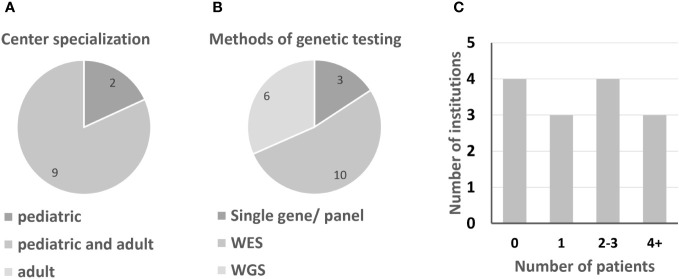
Information on German IEI centers. **(A)** Center specialization for pediatric or adult IEI patients. **(B)** Preferred molecular genetics; multiple answers were possible. **(C)** Number of patients per institution, clustered.

From a pool of 40 potential interviewees, 15 agreed to answer the questionnaire (see supplements) and to participate in a subsequent interview. Participants are employed in immunological centers and clinics in Berlin, Dortmund, Dresden, Frankfurt, Freiburg, Giessen, Greifswald, Hannover, Leipzig, Munich, Siegen and Würzburg. Due to the rarity of APDS, some of the 40 initial interview candidates had not heard of APDS and many were aware of the disease but had never seen a patient. Of the 15 participants, 67% indicated having a background in pediatrics, while 33% treat adult patients (**Figure 1**, question 1a). 47% are specialists for haemato-oncology, 40% for rheumatology and 27% for pulmonology (question 1, multiple answers were possible). To clarify open questions that remained after the initial round of interviews, we prepared a second questionnaire (see follow-up questionnaire in supplements), to which seven participants from the initial interview series responded.

### IEIs: diagnostic landscape in Germany, location of APDS patients

Due to the rarity of IEI, patients are ideally diagnosed and treated in centers specialized in immunological diseases. Most institutions treat both adult and pediatric patients (9 out of 11 institutions treat both, while 2 centers specialize in pediatric immunology). Usually responsibilities for the two age groups are located in different departments. According to the interviewees, especially from smaller institutions, the care of adolescents and young adults by pediatricians may be continued into mid-adulthood. Between the centers in Germany, there are significant differences in terms of capacity for immunological patients (questions 4, 4a). The number of APDS patients who have been or are being treated at the respective centers hardly correlates with their capacity (question 5). Half of all centers currently treat only one patient or none (**Figure 1**; **Table 1**). As expected, APDS occurs in clusters, i.e. over several generations.

**Table 1:** Patient capacity of centers in this study, per estimate by interviewees.

Center/clinic	Pediatric or adult patients?	Immunodeficient patients total	combined and B-cell deficiencies	APDS patients
Berlin (Charité - Institut für Medizinische Immunologie/ Immundefekt-Ambulanz)	adult	500	400	1*
Berlin (Charité Universitätsmedizin – Klinik für Pädiatrie)	pediatric	300	100	5*
Dortmund (Klinikum Dortmund (Universitätsmedizin Witten) – Kinderonkologisches Zentrum	pediatric	–	–	0
Dresden (UniversitätsCentrum für Chron. Immundefizienzen, Universitätsklinikum CGC Dresden – Klinik und Poliklinik für Kinder- und Jugendmedizin/Medizinische Klinik 1)	pediatric	250	130	3*^#^
Frankfurt (Universitätsklinikum Frankfurt – Klinik für Kinder- und Jugendmedizin)	pediatric	200	100	0
Freiburg (Universitätsklinikum Freiburg – Centrum für Chronische Immundefizienz)	adult/pediatric	1000	850	5*
Gießen (Universitätsklinikum Gießen - Klinik für Kinder- und Jugendmedizin)	pediatric	80	50	1*
Greifswald (Klinik und Poliklinik für Kinder und Jugendmedizin)	pediatric	30	15	0
Hannover (MHH - Klinik für Rheumatologie & Immunologie/Immunologische Ambulanz II/Infektiologie und Immundefizienzen)	adult	650	600	2
Hannover (MHH – Klinik für Pädiatrische Pneumologie, Allergologie und Neonatologie)	pediatric	141	75	4
Leipzig (Universitätsklinikum Leipzig – Klinik und Poliklinik für Kinder- und Jugendmedizin)	pediatric	45	–	0
Munich (LMU - Dr. von Haunersches Kinderspital Kinderklinik und Kinderpoliklinik)	pediatric, adult	550	180	3^#^
Siegen (St. Marien-Krankenhaus – Institut für Klinische Immunologie)		–	326	3
Würzburg (Universitätsklinikum Würzburg – Klinik für Kinder- und Jugendmedizin)	pediatric	150	100	1*

*one patient received hematopoietic stem cell transplantation; ^#^one patient died (not included in the count). CID, combined immunodeficiency; CGC, Carl-Gustav-Carus; LMU, Ludwig-Maximilians-Universität; MHH, Medizinische Hochschule Hannover.

**Figure 2:**
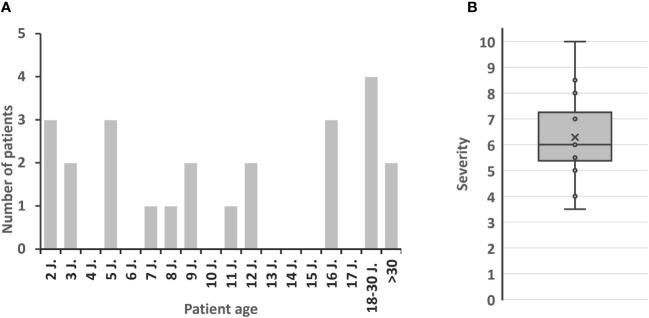
Patient characteristics. **(A)** Age at APDS diagnosis. **(B)** Estimated severity of APDS by interviewees (1 = well symptomatically treatable to 10 = immediately life-threatening, indication for HSCT); x = mean value.

A total of 28 patients with APDS was reported by participants of our survey. Of 26 patients in whom the APDS subtype was known, 17 were diagnosed with APDS1 and 9 with APDS2 (question 8), which is in line with reports in literature (28, 29). At the age of diagnosis, 15 out of 24 patients were 12 years old or younger. Six patients were diagnosed at an age of >18 years, with two being diagnosed at 30 or later (question 9, **Figure 2**). Interestingly, investigation of first-degree relatives revealed carriers of APDS-associated variants, some of whom were oligo- or asymptomatic. Asymptomatic variant carriers not considered and treated as APDS patients were not included in this analysis. This phenomenon in autosomal dominant IEI such as APDS is known as ‘reduced penetrance’. IEI with variable penetrance are a challenge to the clinician managing these individuals.

The ESID APDS registry study (30) is a project that aims to collect data on the natural clinical course of APDS patients and their treatments. Currently, 28 APDS patients from Germany are registered, which relative to population size is less than numbers of registered patients in France (n=53), the UK (n=34), the Czech Republic (n=7) and Switzerland (n=6) (30, 31). Nine participants in our survey have included their patients in the registry, while six did not (question 12). The most common reasons for non-participation were lack of consent from patients and time constraints. Some participants indicated that support for data collection and data recording could increase participation in the registry.

### Genetic testing of IEI patients

Genetic testing has become part of the standard diagnostic procedure in suspected IEI patients. 40% of interview participants reported performing genetic testing in-house, while 60% had patient samples sequenced externally (question 10, 10a). External testing was performed primarily at two national sites: MH Hannover and CEGAT Tübingen. No participant reported exclusively using gene panels, but 13% still opt for panels occasionally. Use of whole exome sequencing (WES) was most commonly reported (60%), followed by whole genome sequencing (WGS), with 40%. WGS in particular has become more available in recent years. In clinical practice, data from WES and WGS are rarely analyzed at large for reasons of practicability, human resources, and reimbursement issues. Instead, so-called virtual panels are performed, in which only a subset of known IEI-associated genes is analyzed, similar to traditional gene panels. Several participants however reported performing analysis of the complete exome or genome as part of academic research after patient consent. Once diagnosis of a specific IEI is established in an individual patient, family members may be tested for segregation or carrier status using Sanger sequencing of the candidate gene (question 11).

Today, newborn screening for severe T-cell lymphopenia/severe combined immunodeficiency (SCID) is recommended by many national guidelines to avoid diagnostic delay. As part of our interviews, we asked whether newborn screening should be extended to IEI of moderate to high severity (questions 13, 13a). 70% of participants agreed. Disagreement was based on ethical concerns, potential reimbursement issues, and culturally based widespread reservations about routine genetic testing. When asked to specify diseases which should be screened for, chronic granulomatous disease (CGD, n=4 interviewees) and hemophagocytic lymphohistiocytosis (HLH, n=3) were indicated most frequently. Three participants mentioned APDS, while other participants emphasized that they would not test for APDS, since the clinical course is highly variable and diagnosis at birth would not immediately influence treatment decisions. These current expert opinions may illustrate the limitations of the “genotype-first” approach. Nonetheless, all participants stated that the availability of targeted therapy could make early genetic testing in some IEI more relevant in the future and *vice versa* (question 25b).

### Clinical picture and diagnosis of APDS

APDS is a monogenic IEI, presenting with a spectrum of signs and symptoms, and has a large phenotypic variability from mild to severe. On a scale from 1-10, where 1 is “well symptomatically treatable” and 10 is “immediately life-threatening, indication for HSCT”, most participants of this survey would classify their patients between 5 and 7.5 (average: 6, range: 3.5-10) (question 19; **Figure 2**). Several participants (n=6/15) indicated variable degrees of severity, which reflects the heterogeneity of patients, and acknowledges the fact that each physician’s expertise and perception of APDS is greatly influenced by the clinical picture of very few patients under their care. Lymphoproliferation and susceptibility to infection are usually the earliest signs of APDS (32). In line with data from the literature APDS patients tend to first present with frequent infections (n=7/7 respondents of the follow-up questionnaire) or lymphoproliferation (lymphadenopathy and/or hepatosplenomegaly, n=6/7). They next reported hypogammaglobulinemia (n=5/7) and immune dysregulation (n=4/7) affecting the GI tract (colitis), joints (arthritis) and hematopoietic system (cytopenia), and developmental disorders. (**Question S1**, **Table S1**). Microcephaly and/or mild intellectual deficiency occurred exclusively in APDS2 patients (**Question S12**). No severe courses of COVID-19 were reported (**Question S11**).

The survey next asked participants to indicate features which lead the diagnosing physician to suspect APDS in each of their previous patients (question 6). Participants indicated a variety of symptoms and features (multiple mentions were possible, see Methods section): infection susceptibility (9 mentions), benign lymphoproliferation (7 mentions), immune dysregulation (6 mentions) and laboratory parameters (5 mentions). Developmental disorders (1 mention) and lymphoma (2 mentions) were cited less frequently (details in **Table S2**). In a later question we asked which symptoms each expert would consider most indicative of APDS, given their current knowledge (Question 14). Pathognomonic laboratory features were cited as highly indicative of APDS, including humoral (high IgM + low IgG, 9 mentions in total) and immune phenotyping reminiscent of immune dysregulation in APDS (13 mentions in total), susceptibility to infections (20 mentions), immune dysregulation (11 mentions) and benign lymphoproliferation (10 mentions). Malignant lymphoma was cited twice (2 mentions; detailed breakdown in **Table S3**). Two participants reported on APDS patients diagnosed only after developing lymphoma which underscores the reality of diagnostic delay.

Despite the improved availability of genetic testing, clinical and immunological expertise continues to be key to the timely diagnosis of patients with IEI. Most participants in this survey indicated that descriptive and functional immune diagnostics would usually precede genetic testing. The definite diagnosis of APDS is then established after identification of a relevant APDS-associated *PIK3CD* or *PIK3R1* variant. In line with data from literature, the E1021K variant in *PIK3CD* was by far the most frequent finding in APDS1 patients, whereas all APDS2 patients had a splice site mutation in *PIK3R1*. Multiple interview partners reported patients who presented with a disease pattern resembling APDS, but inconclusive genetics. Experts suggested to establish a validated functional assay to test for PI3Kδ/Akt activity in order to better determine the biological impact of *PIK3CD* or *PIK3R1* gene variants in individual phenotypes. Such an assay could analyze phosphorylation levels of proteins downstream of PI3Kδ, including Akt, FOXO1, and the ribosomal protein S6 (10, 13, 33).

### Management of APDS patients

Via a second questionnaire, we asked participants how often APDS patients would come for follow-up appointments, and how they were monitored and treated. Frequency of follow-ups differed between once (n=2/7 respondents) to four times (n=5/7) per year. Nearly all APDS patients had received or were currently under antibiotic treatments (Tobramycin, ceftazidime, azithromycin, amoxicillin, cotrimoxazole, and combinations thereof) and IRT (mostly subcutaneously). Three participants indicated use of rapamycin and two participants reported using rituximab in the past. With the exception of one patient, rapamycin therapy had been discontinued (1x due to lack of efficacy, 1x discontinuation post-HSCT).

Of 28 patients currently or formerly followed by participants of this survey, 6 were post-HSCT. Participants estimated one third of APDS patients to be eligible for HSCT who were perspectively planned for this treatment (question 18). Attitudes towards HSCT differed among interview partners. Most considered it a viable option, some even the best option for most patients with severe symptoms. A few, however, considered it to be too risky for most APDS patients. Some expressed their concern about the efficacy of stem cell transplantation in IEI patients with GOF gene variants in general. GOF variants in autologous cells present post-HSCT could have a growth advantage over wild-type donor cells.

Leniolisib, which is a PI3Kδ-specific inhibitor, could be a treatment with low risk of severe side effects to be given lifelong on a daily basis. Based on opinions and suggestions by multiple participants, the targeted drug treatment may suffice in patients with mild and moderate disease symptoms. Alternatively, leniolisib may be used as a bridging therapy until HSCT. All experts were favorable of this targeted therapy and were open to using leniolisib in a compassionate use program (questions 20, 21). Most respondents to the follow-up questionnaire (n=4/7) would use leniolisib for all APDS patients if available. Others would turn to leniolisib only in case of severe lymphoproliferation or immune dysregulation (n=2/7) (question S8). When asked about which parameters were useful to monitor efficacy of this targeted therapy (question 22; multiple answers were possible), most participants referred to immunophenotyping with normalization of T- and B-cell alterations (14 mentions), resolution of lymphoproliferation (13 mentions) and susceptibility to infections (12 mentions). Effects on immune dysregulation (3 mentions), developmental disorder (3 mentions), quality of life (4 mentions) and lymphoma risk (1 mention) were cited less frequently (details **Table S4**). When asked what targeted therapy would have to achieve to replace HSCT (question 23), experts would rather stress quality of life (QoL)-related parameters (10 mentions) and susceptibility to infection (9 mentions). Laboratory parameters (4 mentions) and benign lymphoproliferation (5 mentions) were also considered, as were effects on immune dysregulation and developmental disorder (2 mentions each). However, lowering the risk of lymphomagenesis (4 mentions) was deemed more important if lifelong targeted therapy were favored over HSCT (details in **Table S5**). The 3-year safety data from leniolisib were considered reassuring, still some experts would be hesitant of prescribing an immunomodulatory drug for life. Amongst the survey participants, some had experience with leniolisib for their patients in a compassionate use program. While satisfaction with available treatment options was mixed, ranging from very low to high satisfaction (question S4), physicians who had already used leniolisib were satisfied with its disease-modulating activity and safety (question S5). One respondent reported safety and tolerability issues with prednisolone (arterial hypertension, kidney insufficiency, osteonecrosis) and cyclosporin (infections). The other respondents reported acceptable to good tolerability of the chosen treatment options, including rapamycin and leniolisib. Several respondents reported being able to discontinue other medications (e.g., antibiotics) in their patient after starting therapy with leniolisib. One single APDS patient had become symptom-free under treatment with leniolisib. For another patient the molecule had a significant effect on lymphoproliferation, while it did not reduce their susceptibility to infection.

### Challenges in IEI diagnosis

Most experts consulted in this study are from centers dedicated to diagnosing and treating IEI patients. The main difficulty for non-immunologists - general practitioners, pediatricians, and specialists of other fields - to diagnose IEI patients, however, is to recognize disease patterns hinting at these rare diseases and to refer the patient to an IEI center. In our questionnaire, we asked, whether training non-immunologists working outside of IEI centers would help raise awareness of IEI including APDS (question 16). Most of the interviewees (87%) were optimistic about the efficacy of such training, but others were anxious about aligning the contents to the need of a diverse audience. The teaching of relevant content, especially that which is necessary for the recognition of IEI, should be achieved without having to teach immunological concepts in an exhaustive manner. For this purpose, training initiatives should start by informing about warning signs for IEI in general before going into specific IEI such as APDS. The warning signs could include the so-called ELVIS and GARFIELD criteria detailed in the German guideline on diagnostics in IEIs (8) or those suggested in the US guidelines (7). Teaching could include discussion of instructive case studies to illustrate the IUIS classification of IEI (3) and convey the first diagnostic steps and pitfalls in detecting patients with undiagnosed IEI in trainings, each of which are specifically geared to one specialist group. Depending on the subspecialty, case reports for e.g. hemato-oncologists would emphasize the co-existence of IEI and immune thrombocytopenia, the occurrence of malignant lymphomas in various IEIs, or else the challenge involved in treating lymphoma patients with an underlying IEI. Ideally, the trainings could be regularly adapted according to the latest state of knowledge, which could be implemented especially well with non-linear media.

## Conclusion

As is the case for rare diseases in general, diagnosis and treatment of IEI in Germany continues to challenge caretakers. Lack of awareness of IEI among pediatricians and specialists outside of IEI centers is the rule. The heterogeneity of symptoms and features of many IEIs - including APDS - further complicates the situation. In order to improve this situation, more pediatricians should familiarize themselves with the warning signs and clinical cues of IEI. This also holds true for other subspecialties where IEI patients may first present, as genetic diagnostics are becoming increasingly available. Nevertheless, precise genetic diagnosis, thorough interpretation of novel genetic variants or variants in genes not yet known for causing IEI, remain a challenge to clinicians and geneticists alike. Functional testing of patient primary cells could direct the identification of affected pathways and molecular causes in suspected monogenic IEI. Furthermore, such assays could guide the selection of appropriate therapies and facilitate treatment monitoring, which is central given the increasing number of targeted therapies available.

For the treatment of APDS, two main therapeutic options are available besides supportive management – HSCT and targeted therapy. While the former ideally represents a one-time intervention, it carries significant treatment-related risks. Treatment with mTOR inhibitors or leniolisib improve the quality of life for many patients with a calculated risk profile. The disadvantage is lifelong treatment, medication side effects, and possibly refractory disease or progression over time. Also, long-term outcome data under targeted therapies is currently limited (26). According to most of the survey participants, a significant improvement in quality of life would have to be achieved with a targeted therapy to dispense with HSCT permanently, and also, therapeutic benefits should override possible medication side effects. From the perspective of participating experts, the use of both treatment options is compatible with real life practice. HSCT or targeted therapy may be used in different patients, or different disease settings. For example, HSCT could be used for younger patients expected to have a more severe course, while a targeted drug treatment may suffice in patients with less severe disease-associated symptoms (29). In addition, patients who would generally be well suited for HSCT but are in poor clinical condition could first be stabilized with an PI3Kδ inhibitor such as leniolisib. Adolescent and adult patients (e.g., aged 18+) with low suitability for HSCT, or patients with graft failures could be considered for lifelong treatment. By identifying underlying molecular mechanisms, targeted therapies could prospectively gain in importance. Compared to the era of symptom-oriented treatment, easier access to exome and genome sequencing in the current era may inform therapeutic decisions more readily.

APDS is a very rare disease, which is why the number of physicians with experience in treating patients with APDS is quite low. The low number of participants, especially in the follow-up questionnaire therefore represents a limitation for the APDS-specific statements. We suggest that it would be important to extend this kind of survey to other European centers to create a broader data base and to train non- immunologists working outside of IEI centers to help raise awareness of IEI including APDS.

## Methods

### Design of the questionnaire

The questionnaire was developed in a joint effort by the authors with the goal of assessing the diagnostic and treatment landscape in IEI and specifically APDS. It covers the following topics: Structural aspects of the German health care system regarding IEI, diagnostic procedures for IEI patients, distribution of APDS patients, APDS symptoms and severity, treatment pathways in APDS, role of HSCT and targeted therapy in APDS, influence of molecular diagnostics on targeted therapies. Symptoms and indicators of disease were classified into the following groups: Laboratory parameters (e.g., antibody subclass deficiency, immune cell counts), benign lymphoproliferation, malignant disease, susceptibility to infection, developmental disorders, immune dysregulation/autoimmunity, and quality of life parameters (including adverse effects from treatment).

### Selection of the interview partners

Interview partners were chosen based on their expertise at a clinic or center specializing in the management of IEI patients. All participants have had experience in the treatment of patients with IEI at their respective institutions or in cooperation with other institutions.

### Survey process

The questionnaire was distributed in German language to experts, who were given at least one week to answer the survey. In addition, individual interviews were conducted with all participants to clarify open issues relating to topics contained in the questionnaire.

### Follow-up questionnaire

After completion of the interview process, important open questions and missing data points were collected and compiled into a second questionnaire that was sent out to those participants who had previous experience with APDS patients. Seven experts responded to the follow-up questionnaire.

### Data processing

Statements and opinions of the interviewees were processed individually and the contents were compiled in this publication in a non-personalized fashion. Data were analyzed quantitatively where possible.

Symptoms and features described by respondents were consolidated as distinct items and then grouped into categories at the discretion of the authors (e.g., ‘low IgG’ and ‘hypogammaglobulinemia’ were consolidated as ‘hypogammaglobulinemia’; hypogammaglobulinemia was then counted as a subset of ‘laboratory parameters’). Multiple features could be indicated by each participant on each question. Each reported symptom or feature was counted as one mention.

## Data availability statement

The original contributions presented in the study are included in the article/**Supplementary Material**. Further inquiries can be directed to the corresponding authors.

## Author contributions

SV: Conceptualization, Data curation, Formal Analysis, Investigation, Methodology, Visualization, Writing – original draft. BG: Conceptualization, Methodology, Supervision, Validation, Writing – review & editing, Resources. FH: Resources, Validation, Writing – review & editing. AM: Resources, Validation, Writing – review & editing. GS: Resources, Validation, Writing – review & editing. LH: Resources, Validation, Writing – review & editing. MM: Resources, Validation, Writing – review & editing. CS: Resources, Validation, Writing – review & editing. JK: Writing – review & editing.

## Supplementary material

Supplementary Table 1 Symptoms of APDS patients at presentation (**Question S1**). Data based on follow-up questionnaire with seven respondents. Number of mentions in brackets (multiple signs/symptoms could be named). EBV, Epstein-Barr virus; HPV, human papilloma virus; HSV, Herpes simplex virus; n.s., non-specified; RTI, respiratory infection.

Supplementary Table 2 Symptoms leading to APDS diagnosis (question 6). Number of mentions in brackets (multiple signs/symptoms could be named). n.s., non-specified.

Supplementary Table 3 Symptoms most indicative of APDS (question 14). Number of mentions in brackets (multiple signs/symptoms could be named). CID, combined immunodeficiency; CVID, Common variable immunodeficiency; EBV, Epstein-Barr virus.

Supplementary Table 4 Useful parameters to indicate efficacy of a targeted therapy in APDS (question 22). Number of mentions in brackets (multiple signs/symptoms could be named). 6MWT, 6-minute-walking-test; BMI, body mass index; EBV, Epstein-Barr-virus; FEV1; Forced expiration volume in 1 second; n.s., non-specified; RTI, respiratory tract infections.

Supplementary Table 5 Therapy goals which would have to be achieved with targeted therapy to dispense with HSCT permanently (question 23). Number of mentions in brackets (multiple signs/symptoms could be named). CR, complete response; EBV, Epstein-Barr virus; n.s., non-specified; PR, partial response.
