# Supplementary material for: Future Directions in the Diagnosis and Treatment of APDS and IEI: a Survey of German IEI Centers
Source: Front Immunol. 2023 Oct 5;14:1279652. doi: 10.3389/fimmu.2023.1279652 (PMC10588788; doi:10.3389/fimmu.2023.1279652)
Supplement: Supplementary Table 1 — Symptoms of APDS patients at presentation ( Question S1 ). Data based on follow-up questionnaire with seven respondents. Number of mentions in brackets (multiple signs/symptoms could be named). EBV, Epstein-Barr virus; HPV, human papilloma virus; HSV, Herpes simplex virus; n.s., non-specified; RTI, respiratory infection. [file Table_1.docx]

# Supplements

## Tables

| **Table S1: Symptoms of APDS patients at presentation (Question S1).** Data based on follow-up questionnaire with seven respondents. Number of mentions in brackets (multiple signs/symptoms could be named). EBV, Epstein-Barr virus; HPV, human papilloma virus; HSV, Herpes simplex virus; n.s., non-specified; RTI, respiratory infection. | | |
| --- | --- | --- |
| **Laboratory parameters (5)** | **Infection susceptibility (12)** | **Immune dysregulation (6)** |
| Hypogammaglobulinemia (5) | Bronchiectases (4) | Granulomatous gut inflammation (1) |
| **Developmental disorders (5)** | EBV (1) | Gastrointestinal complaints, n.s. (1) |
| Neurological disorder, n.s. (1) | Recurring RTI (2) | Arthritis (1) |
| Microcephaly (1) | HPV (1) | Colitis (1) |
| Premature aging (1) | HSV (1) | Immune thrombocytopenia (1) |
| Facial dysmorphia in APDS2 (1) | Recurring infections, n.s. (1) | Autoimmune hemolytic anemia (1) |
| Short stature (1) | Bacterial bronchitis (1) | **Benign Lymphoproliferation (11)** |
|  | Otitis media (1) | Lymphadenopathy (5) |
|  |  | Spleno-/Hepatomegaly (6) |
