# Supplementary material for: Future Directions in the Diagnosis and Treatment of APDS and IEI: a Survey of German IEI Centers
Source: Front Immunol. 2023 Oct 5;14:1279652. doi: 10.3389/fimmu.2023.1279652 (PMC10588788; doi:10.3389/fimmu.2023.1279652)
Supplement: Supplementary Table 2 — Symptoms leading to APDS diagnosis (question 6). Number of mentions in brackets (multiple signs/symptoms could be named). n.s., non-specified. [file Table_2.docx]

# Supplements

## Tables

| **Table S2: Symptoms leading to APDS diagnosis (question 6).** Number of mentions in brackets (multiple signs/symptoms could be named). n.s., non-specified. | | |
| --- | --- | --- |
| **Laboratory parameters (5)** | **Infection susceptibility (9)** | **Immune dysregulation (6)** |
| Hypogammaglobulinemia (4) | Pneumonia | Arthritis (1) |
| Immune cell cytopenia (1) | Recurrent respiratory tract infections (2) | Autoimmune hemolysis (1) |
| Developmental disorders (1) | Recurrent pulmonary infections (2) | Autoimmune cytopenia (1) |
| **Malignant disease (2)** | Cough/ Bronchiectases (2) | Rheumatoid symptoms (1) |
| Lymphoma (2) | **Benign Lymphoproliferation (7)** | Crohn’s-like disease (1) |
| **Developmental disorders (1)** | Lymphadenopathy (7) | Immune dysregulation, n.s. (1) |
| Failure to thrive (1) |  |  |
