# Supplementary material for: Future Directions in the Diagnosis and Treatment of APDS and IEI: a Survey of German IEI Centers
Source: Front Immunol. 2023 Oct 5;14:1279652. doi: 10.3389/fimmu.2023.1279652 (PMC10588788; doi:10.3389/fimmu.2023.1279652)
Supplement: Supplementary Table 3 — Symptoms most indicative of APDS (question 14). Number of mentions in brackets (multiple signs/symptoms could be named). CID, combined immunodeficiency; CVID, Common variable immunodeficiency; EBV, Epstein-Barr virus. [file Table_3.docx]

# Supplements

## Tables

| **Table S3: Symptoms most indicative of APDS (question 14).** Number of mentions in brackets (multiple signs/symptoms could be named). CID, combined immunodeficiency; CVID, Common variable immunodeficiency; EBV, Epstein-Barr virus. | |
| --- | --- |
| **Infection susceptibility (20)** | **Laboratory parameters (22)** |
| Recurrent respiratory tract infections (6) | Immune cytopenia (5) |
| Bronchiectases (6) | CID (1) |
| Chronic EBV-Infection (6) | Hypogammaglobulinemia (5) |
| CVID phenotype (1) | Hyper IgM (4) |
| Cobblestone aspect of bronchial mucosa (1) | CD4+ T-cell lymphocytopenia (1) |
| **Immune dysregulation (11)** | High CD57+ T-cell count (1) |
| Autoimmunity (4) | Low level of naive T cells (1) |
| Gastrointestinal symptoms, colitis (5) | Increased transitional B cells (3) |
| Subclinical inflammation (1) | Lowered class-switched B cells (1) |
| Autoimmune cytopenia (2) | **Benign lymphoproliferation (10)** |
| **Malignant disease (2)** | Lymphadenopathy (10) |
| Lymphoma (2) |  |
