# Supplementary material for: Future Directions in the Diagnosis and Treatment of APDS and IEI: a Survey of German IEI Centers
Source: Front Immunol. 2023 Oct 5;14:1279652. doi: 10.3389/fimmu.2023.1279652 (PMC10588788; doi:10.3389/fimmu.2023.1279652)
Supplement: Supplementary Table 4 — Useful parameters to indicate efficacy of a targeted therapy in APDS (question 22). Number of mentions in brackets (multiple signs/symptoms could be named). 6MWT, 6-minute-walking-test; BMI, body mass index; EBV, Epstein-Barr-virus; FEV1; Forced expiration volume in 1 second; n.s., non-specified; RTI, respiratory tract infections. [file Table_4.docx]

# Supplements

## Tables

| **Table S4: Useful parameters to indicate efficacy of a targeted therapy in APDS (question 22).** Number of mentions in brackets (multiple signs/symptoms could be named). 6MWT, 6-minute-walking-test; BMI, body mass index; EBV, Epstein-Barr-virus; FEV1; Forced expiration volume in 1 second; n.s., non-specified; RTI, respiratory tract infections. | | |
| --- | --- | --- |
| **Laboratory parameters (14)** | **Infection susceptibility (12)** | **Immune dysregulation (3)** |
| Immune exhaustion (1) | EBV infections (2) | Autoimmunity (2) |
| CD4 T-cell lymphocytopenia (1) | Infection susceptibility, n.s. (4) | Immune dysregulation, n.s. (1) |
| IgG and IgM levels (2) | Frequency/ quality of cough (1) | **Developmental disorders (3)** |
| Normalization of T- und B- cell alterations (7) | FEV_1_ (1) | Thriving (BMI, Z-Score) (2) |
| Normalization of increased transitional B-cell levels (3) | Viral load (1) | Delayed longitudinal growth (Z-Score) (1) |
| **Benign lymphoproliferation (13)** | Regression of RTIs (1) | **Quality of life (4)** |
| Lymphadenopathy (11) | Cobblestone aspect of bronchial mucosa (1) | Possibility of discontinuing other therapies (1) |
| Organomegaly/ lymphadenopathy (2) | Exacerbation of RTIs (1) | Better 6MWT (older patients) (1) |
| **Malignant disease (1)** |  | Control of clinical manifestations without toxicity (1) |
| Lymphoma (1) |  | Significant symptom reduction (1) |
