# Supplementary material for: Future Directions in the Diagnosis and Treatment of APDS and IEI: a Survey of German IEI Centers
Source: Front Immunol. 2023 Oct 5;14:1279652. doi: 10.3389/fimmu.2023.1279652 (PMC10588788; doi:10.3389/fimmu.2023.1279652)
Supplement: Supplementary Table 5 — Therapy goals which would have to be achieved with targeted therapy to dispense with HSCT permanently (question 23). Number of mentions in brackets (multiple signs/symptoms could be named). CR, complete response; EBV, Epstein-Barr virus; n.s., non-specified; PR, partial response. [file Table_5.docx]

# Supplements

## Tables

| **Table S5: Therapy goals which would have to be achieved with targeted therapy to dispense with HSCT permanently (question 23).** Number of mentions in brackets (multiple signs/symptoms could be named). CR, complete response; EBV, Epstein-Barr virus; n.s., non-specified; PR, partial response. | | |
| --- | --- | --- |
| **Infection susceptibility (9)** | **Quality of life (10)** | **Laboratory parameters (4)** |
| EBV infections (1) | Quality of life, not specified (2) | Immune Exhaustion (1) |
| Infection susceptibility, n.s. (5) | Tolerability of therapy (1) | Polyclonal naive T-cell repertoire w/o significant adverse effects (1) |
| Avoidance of severe systemic infection (1) | Significant reduction of symptoms (4) | Improvement of CD4+ T-cell lymphocytopenia (1) |
| Viral load (1) | Control of clinical manifestations without toxicity (2) | Immunological parameters (Igs, cell counts) (1) |
| Bronchopulmonary infection (1) | Stable PR or CR with good tolerability (1) | **Benign Lymphoproliferation (5)** |
| **Developmental disorders (2)** | Possibility of discontinuing other therapies (1) | Lymphoproliferation (4) |
| Improvement of failure to thrive (1) | **Lymphoma (4)** | Organomegaly/ lymphadenopathy (1) |
| Normal growth (1) | Prevention of lymphoma (3) | **Immune dysregulation (2)** |
|  | Reduction of lymphoma risk (1) | Autoimmunity (2) |
