# Supplementary material for: Future Directions in the Diagnosis and Treatment of APDS and IEI: a Survey of German IEI Centers
Source: Front Immunol. 2023 Oct 5;14:1279652. doi: 10.3389/fimmu.2023.1279652 (PMC10588788; doi:10.3389/fimmu.2023.1279652)
Supplement: Supplementary file 6 [file DataSheet_1.docx]

# Supplements

## Questionnaire

Note that in the questionnaire, we used the term Primary Immunodeficiency (PID) instead of IEI.

1. What is your specialization (immunology, pulmonology, (hemato-)oncology, infectiology, rheumatology, intensive care, other)?
   1. Do you work in pediatric or adult medicine?
2. What capacity for patients does your facility have?
3. Does your facility have specialty outpatient clinics for pediatric or adult PID patients or both?
4. What is the total number of PID patients currently being treated at your facility?
   1. Of these, how many patients have combined immunodeficiencies/leading AK deficiency?
5. How many patients with APDS are you aware of and how many are currently being treated at your institution?
6. How was APDS diagnosed in each case (leading symptoms)?
7. What specialties do the diagnosing physicians have (see question 1)?
8. Which APDS variants were diagnosed?
9. How old were the APDS patients at the time of diagnosis?
10. Do you have genetic testing performed internally or externally to diagnose PIDs?
    1. If external, where?
11. What genetic diagnostics do you use (WES, WGS, panel, ...)?
12. Do you participate in the ESID registry study on APDS?
13. Nowadays, newborns are routinely tested for SCID/severe T-cell lymphopenia. Should routine genetic testing be expanded to include other less severe PIDs if relevant to treatment decisions? (y/n)
    1. If yes, do you have specific PIDs in mind?
14. Is there a symptom or symptom combination that makes you think of APDS and if so, which one(s) (laboratory parameters, respiratory infections, bronchiectases, cytopenias, autoimmunity, gastrointestinal symptoms, lymphoproliferation, chronic EBV infections, other)?
15. Have you experienced developmental delays or growth retardation in your APDS cases (y/n)?
    1. If yes, were these cases diagnosed with APDS rather later (after 12 years of age)? (y/n)?
16. APDS was first described as a distinct PID in 2013 and only a few physicians outside of PID centers know of cases from personal experience. Do you see a need for trainings in this area?
17. If patients with hematologic malignancies also have PID, this can affect the available therapies. Do you routinely test young patients with, for example, lymphoma for PIDs? (y/n)
    1. If not, as a PID expert, are you often called to test for undiagnosed PID in hematologic oncology patients? (y/n)
18. What percentage of APDS patients undergo hematopoietic stem cell transplantation (HSCT) sooner or later?
19. If you compare APDS to other forms of PIDs, where would you rank it on a scale of 1 ="well symptomatically treatable (i.e., antibiotics, IRT, immunosuppressants)" to 10 ="immediately life-threatening, indication for HSCT" (such as SCID)?
20. Do you consider blockade of the PI3Kδ-pathway, e.g. with leniolisib, as a reasonable approach for therapy of the cause of APDS (y/n)?
21. Would you consider using leniolisib in APDS patients now if it were available in a compassionate use program? (y/n/only after publication of phase III data).
22. Which target parameters or endpoints do you consider appropriate to demonstrate the efficacy of targeted therapy in APDS?
23. Which therapeutic goal would have to be achievable with leniolisib in order to permanently avoid HSCT?
24. Do you use questionnaires to assess quality of life in your patients and if yes, which ones?
25. Do you think that targeted drugs like leniolisib can significantly improve the lives of PID patients in the future (y/n)?
    1. (If yes) With the availability of targeted therapies for certain forms of PID, will early differential diagnosis, including genetic diagnosis, become more important? (y/n)

## Follow-up questionnaire

1. Which symptoms did APDS patients presented with to you?

- Lymphoproliferation
- Spleno-/hepatomegaly
- Brochiectases
- Gastrointestinal complaints
- Recurrent infections; EBV/CMV; Other
- Hypogammaglobulinemia
- Immune dysregulation (which one?)
- Other symptoms

1. On average, how often do you see your APDS patients in your consultation?

- Regularly 1x per quarter
- Regularly every 6 months
- 1x per year
- When needed

1. How are your APDS patients currently being medicated? Are you aware of patients’ treatment prior to APDS diagnosis?

- mTOR inhibition
- IRT (subcutaneous or intravenously)
- Antibiotics (if so: which one?)
- Rituximab
- Other (which one?)

1. How satisfied are you with current therapy options? (scale 1 [not satisfied] to 5 [highly satisfied])
2. Could the treatment of individual patients be modified by new treatment options?
3. Are there any side effects of the therapies used that affect the duration of use or dosage?
4. Are there patients you would like to put on therapy with leniolisib now if it were available?
5. For those patients who are currently well: When would you consider using PI3Kδ blockade with leniolisib?
6. In your APDS patients, has the family been co-screened genetically (family history)? (y/n)
7. Which mutation was present?
8. How did your patient(s) survive the SARS-CoV2 infection?
9. Do(es) your patient(s) have any neurological involvement? If yes: which?
